# Ehrlichia infection in Italy.

**DOI:** 10.3201/eid0404.980420

**Published:** 1998

**Authors:** M Nuti, D A Serafini, D Bassetti, A Ghionni, F Russino, P Rombolà, G Macri, E Lillini

**Affiliations:** 1st Rome University, Italy.

## Abstract

Immunoglobulin M seroconversion to Ehrlichia chaffeensis was documented in U.S. citizens bitten by ticks in Sardinia. Seven cases of suspected ehrlichiosis in local residents were not confirmed by laboratory tests. In Alpine areas antibodies to E. phagocytophila were detected in persons at high risk, i.e., foresters (8.6%) and hunters (5.5%), and in controls (1.5%). Of 153 persons bitten by ticks, only one was Ehrlichia antibody-positive after 6 months.


### Ehrlichia Infection in Italy

**M. Nuti,* D.A. Serafini,† D. Bassetti,‡ A. Ghionni,§ F. Russino,¶ P. Rombolà,# G. Macri,# and E. Lillini#**
*1st Rome University, Rome, Italy; †US Navy Medical Services, Colorado Springs, Colorado, USA; ‡Public Health Department, Trento, Italy; §Pieve di Cadore Hospital, Belluno, Italy; ¶Multizone Prevention Service, Belluno, Italy; and #Zooprophylaxis Institute, Rome, Italy

Immunoglobulin M seroconversion to *Ehrlichia chaffeensis* was documented in U.S. citizens bitten by ticks in Sardinia. Seven cases of suspected ehrlichiosis in local residents were not confirmed by laboratory tests. In Alpine areas antibodies to *E. phagocytophila* were detected in persons at high risk, i.e., foresters (8.6%) and hunters (5.5%), and in controls (l.5%). Of 153 persons bitten by ticks, only one was *Ehrlichia* antibody-positive after 6 months.

A newly recognized rickettsial disease, human ehrlichiosis is emerging in tick-infested areas. First described in the United States (1), the disease, in both monocytic and granulocytic forms, is usually found where Lyme disease is endemic (2,3). Sporadic cases of ehrlichiosis have been reported in southern and northern Europe. Recently, cases were reported in Slovenia, not far from the Italian border (4). Preliminary studies show that 17.1%, 5%, 6.3%, and 11.4% of forestry workers in Switzerland, United Kingdom, Italy, and Sweden, respectively (5-8), have antibodies to *Ehrlichia (E. phagocytophila* and *E. equi).*

### Ehrlichiosis in Southern Italy

In 1995 through 1996, four U.S. citizens (two boys and two women) living on a U.S. Navy base in Sardinia (La Maddalena) became ill with acute fever, headache, malaise, and cytopenia after a tick bite. Once rickettsial disease was excluded (Sardinia is a boutonneuse fever–endemic focus), ehrlichia infection was suspected. Coupled serum samples (S1 and S2 a week apart) from each patient were processed by immunofluorescence assay (IFA). All S1 were negative for both species of *Ehrlichia,* while three S2 were positive for immunoglobulin (Ig) M (1:128) to *E. chaffeensis* and negative for IgG (Table), meeting the case definition criteria for ehrlichiosis (Olson, pers. comm.). Subsequently, seven cases of ehrlichiosis-like illness following a tick bite were reported in Sardinian residents. The cases were not confirmed by serologic tests in Rome and Atlanta (E. Lillini, unpub. data).

Table. Immunoglobulin (Ig) M antibodies to *Ehrlichia chaffeensis* in four U.S. citizens bitten by ticks in Sardinia (La Maddalena)

| Case | Date of onset | S1 | No. weeks | S2 |
|---|---|---|---|---|
| 8-yr-old boy[a] | 8/95 | Neg | 1 | 1:128 |
| 12-yr-old boy | 7/96 | Neg | 1 | 1:64[b] |
| 25-yr-old woman | 7/96 | Neg | 1 | 1:128 |
| 24-yr-old woman | 7/96 | Neg | 1 | 1:128[c] |

[a]Evacuated to U.S. Army Hospital in Landsthul, Germany.
[b]Not diagnostically significant.
[c] Third IgM control 1 month later = 1:256.

### Ehrlichiosis in Alpine Areas of Italy

In the last 10 years, tick-borne diseases, especially Lyme disease and tick-borne encephalitis (TBE), have become more widespread in Alpine mountain areas (9). Although new diagnostic tests aid clinical diagnosis, some febrile illnesses in persons bitten by ticks remain unexplained. Human ehrlichiosis must now be considered an emerging problem in tick-infested areas. Pioneering studies in the northern Veneto region (7,10) have recorded *Ehrlichia* antibodies in residents of the Alpine area, where *Ixodes* ticks are present and Lyme disease appears widespread (with more than 500 clinical cases

Address for correspondence: E. Lillini, Istituto Zooprofilattico, Via Appia Nuova 1411, 00178 Roma; fax: 39-6-79340724.

reported annually). This study included Cadore, Bellunese (Veneto region), and Trento Districts, neighboring zones located around an area of 80 to 100 miles in the upper northeastern Alpine region (Figure), which pose equal risk of acquiring tick-borne pathogens.

In 1995 through 1996, 544 serum samples were collected from inhabitants of the northeastern Alpine area presumably at risk for ehrlichiosis because they lived in wooded areas and spent time outdoors. A simplified classification system subdivided the participants according to job or primary vocation: healthy residents (controls, n = 193), rangers and forestry workers from Belluno Regional Park and Forestry Services of Trento District (n = 242), and hunters (n = 109) from both areas. Ehrlichia IgG antibodies were detected by IFA using *E. phagocytophila*–infected ovine neutrophils as antigen (courtesy of Dr. K.J. Sumption, Royal School of Veterinary Studies, Edinburgh University, UK). An IFA titer ≥ 1:80 was considered positive (all analyses contained controls of conjugate antibodies and positive and negative sera). Antibodies to *Borrelia burgdorferi* sensu lato were routinely detected by a commercial standard immunoenzyme test kit (Behring, Marburg, Germany). IgG and IgM were considered positive at values (optical density) of >0.6 for IgG and >0.50 for IgM. The TBE antibody (positive titer ≥ 150) was performed by immunozyme and confirmed by immunoblot (Immuno AG, Wien, Austria). This retrospective serologic study of a cohort of persons at high risk showed an IFA positivity (without benefit of immunoblot confirmation) to

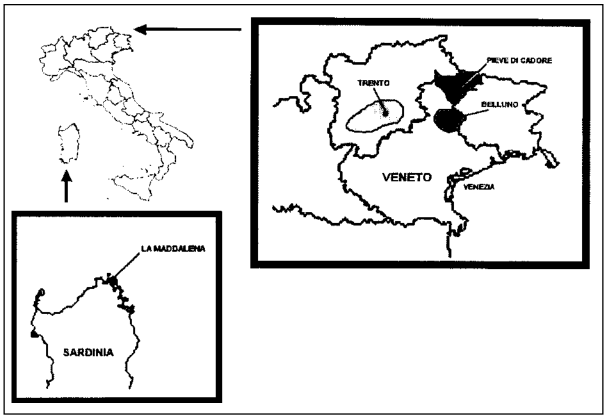

Figure. Areas under study, Sardinia and northeastern Alpine areas.

*E. phagocytophila* (highest titer 1:160) in 21 (8.6%) of 242 foresters, 6 (5.5%) of 109 hunters, and 3 (1.5%) of 193 controls, without significant difference between the three wooded areas studied. Concerning the *Borrelia* positivity, the mean prevalence value among the 541 persons tested was 14.6%, with a lower value (10%) in foresters from Trento District. Seropositivity was most frequent in persons ages 40 to 60 years.

In summer and autumn of 1997, we followed for 6 months 153 Cadore residents bitten by ticks; we detected *Borrelia, Ehrlichia*, and TBE antibodies immediately after the tick bite and 2 and 6 months later. At the preliminary examination, serologic evidence indicated exposure to multiple tick-borne agents in 26 (16.9%) of 153 patients, 15% to *Borrelia*, 0.6% to *E. phagocytophila,* 1.3% to TBE virus. At 2 months, four of the 131 patients tested had IgM antibodies to *Borrelia* (overall incidence 3%); two of these had a clinical pattern of borreliosis. The mean IgG titer to *Borrelia* was 46 (range 7 to >375); the IgM titers were 0.511 to >1.892. Concomitantly, one patient showed typical clinical features of TBE infection, followed by a diagnostic IgM and IgG titer rise (>400); a febrile flulike illness in another patient remained undiagnosed. In the only *E. phagocytophila*–positive person, the titer remained unchanged (1:80). Among the 128 patients tested at 6 months, the four previously recorded as IgM positive to *Borrelia* were confirmed with increased titers (>1,892), and a concomitant IgG positivity (titer >375) was observed in one of these four cases. This study may be one of the largest prospective serologic evaluations for tick-borne pathogens; previous studies have been single-sample serosurveys.

Our data confirm the well-documented high incidence of *Borrelia* infections in upper Alpine areas and show remarkably lower antibody prevalence rates (1%) for TBE and *Ehrlichia*. The different values observed between foresters and healthy controls (8.6% versus 1.5%; p <0.050) from the same zones could be justified by different lifestyles. Foresters spent most of their time in potentially infected areas, changing worksites daily, coming into frequent contact with ticks, and reporting one or more tickbites per year. Controls, on the contrary, had limited outdoor activity, generally restricted to the vicinity of their homes, and less contact with ticks.

Seroconversion to either *Borrelia* or TBE virus were strongly associated with a history of tick attachment; this association and the origin of the persons from a Lyme disease–endemic area support the likelihood that the observed seroconversions were due to a tick-borne agent. The local residents, mainly living in small villages, have close contact with nature but often do not remember tick bites, perhaps because ticks are small and may remain attached for a long time without being noticed. In fact, in approximately 30% of the Lyme disease patients, tick bites are not detectable or reported; consequently, it is difficult to divide these persons into well-defined groups according to tick bite history (9).

Since Lyme disease and ehrlichiosis are zoonoses transmitted by the same tick species *(Ixodes)* and perpetuated in the same reservoir (rodents, deers, sheep), human infections by these pathogens are likely to occur in the same wooded environments. Hunters and rangers, who frequently come in direct contact with animal (deer) carcasses infested by *Ixodes* ticks, or who camp overnight, are at high risk for tick-borne infections (9). Hunters may be infected directly by deer blood (11). *Ehrlichia* seropositivity in persons at risk suggests that mild or subclinical illness due to an infection by *Ehrlichia* species is possible, as recently confirmed (Caruso and Brouqui, pers. data). The low titers observed until now (maximum 1:160) are probably related to a past, mild infection or to the use of an antigen with low specificity.

### Conclusions

Our findings indicate the presence of ehrlichiosis in southern Italy (Sardinia) and of subclinical infections in the upper Alpine northeastern areas. In these forested areas, where most patients and physicians are still unaware of the disease, clinical cases of ehrlichiosis had not been documented, although we can presume that some cases of unexplained febrile illness, especially in areas where Lyme disease is endemic, could be related to *Ehrlichia* infection. Further epidemiologic surveillance and controls, now in progress, may better clarify the emergence of this new infection in Italy.

Dr. Nuti is associate professor of tropical and infectious diseases at the La Sapienza University of Rome. His research has focused on leprosy, viral hepatitis, malaria, dengue, Hantaan virus, and tick-borne diseases.

### References

1. Maeda K, Markowitz N, Hawley RC, Ristic M, Cox D, McDade JE. Human infection with *Ehrlichia canis,* a leukocytic rickettsia. N Engl J Med 1987;316:853-6.
2. Magnarelli LA, Dumler JS, Anderson JF, Johnson RC, Fikrig E. Coexistence of antibodies to tick-borne pathogens of babesiosis, ehrlichiosis, and Lyme borreliosis in human sera. J Clin Microbiol 1995;33:3054-7.
3. Schwartz I, Fish D, Daniels TJ. Prevalence of rickettsial agent of human granulocytic ehrlichiosis in ticks from a hyperendemic focus of Lyme Disease. N Engl J Med 1997;337:49-50.
4. Petrovec M, Furlan SL, Avsic-Zupanc T, Strle F, Brouqui P, Roux V, et al. Human disease in Europe caused by granulocytic *Ehrlichia* species. J Clin Microbiol 1997;35:1556-9.
5. Brouqui P, Dumler JS, Lienhard R, Brossard M, Raoult D. Human granulocytic ehrlichiosis in Europe. Lancet 1995;346:782-3.
6. Sumption KJ, Wright DJM, Cutler SJ, Dale BAS. Human ehrlichiosis in the UK. Lancet 1995;346:1487-8.
7. Lillini E, Macri G, Rombol P, Grazioli D, Russino F, Nuti M. *Ehrlichia* seropositivity in Italian foresters. In: Kazar J, Toman R, editors. Rickettsiae and rickettsial diseases. Bratislava, Slovakia: Slovak Academy of Science; 1996. p. 335-9.
8. Dumler JS, Dotevall L, Gustafson R, Granstrom M. A population-based seroepidemiologic study of human granulocytic ehrlichiosis and Lyme borreliosis on the west coast of Sweden. J Infect Dis 1997;175:720-2.
9. Nuti M, Amaddeo D, Crovatto M, Ghionni A, Polato D, Lillini E, et al. Infections in an Alpine environment: antibodies to hantavirus, leptospira, rickettsiae, and *Borrelia burgdorferi* in defined Italian population. Am J Trop Med Hyg 1993;48:20-5.
10. Nuti M, Russino F, Grazioli D, Rombolo P, Macri G, Lillini E. Anticorpi anti-ehrlichia in soggetti a rischio di zone pedemontane del Veneto. Microbiologia Medica 1996;11:492-5.
11. Bakken JS, Krueth JK, Lund T, Malkovitch D, Asanovich K, Dumler JS. Exposure to deer blood may be a cause of human granulocytic ehrlichiosis. Clin Infect Dis 1996;23:198.

## References

[PDF_00252] Dumler J. S., Dotevall L., Gustafson R., Granström M. (1997). A population-based seroepidemiologic study of human granulocytic ehrlichiosis and Lyme borreliosis on the west coast of Sweden.. J Infect Dis.

[PDF_00227] Maeda K., Markowitz N., Hawley R. C., Ristic M., Cox D., McDade J. E. (1987). Human infection with Ehrlichia canis, a leukocytic rickettsia.. N Engl J Med.

[PDF_00256] Nuti M., Amaddeo D., Crovatto M., Ghionni A., Polato D., Lillini E., Pitzus E., Santini G. F. (1993). Infections in an Alpine environment: antibodies to hantaviruses, leptospira, rickettsiae, and Borrelia burgdorferi in defined Italian populations.. Am J Trop Med Hyg.

[PDF_00245] Sumption K. J., Wright D. J., Cutler S. J., Dale B. A. (1995). Human ehrlichiosis in the UK.. Lancet.

